# Development of sustainable global pediatric surgery: Pioneering solutions from Burkina Faso

**DOI:** 10.1002/wjs.12430

**Published:** 2025-01-15

**Authors:** Sophie Inglin, Anata Bara, Burkina Faso writing group, Yacouba Traoré, Saïd N. Ganame, Abou Coulibaly, Bertille Ki, Seydou Barro, Karim Barro, Emile Bandre, Seni Kouanda, Barbara E. Wildhaber

**Affiliations:** ^1^ Institute of Global Health University of Geneva Geneva Switzerland; ^2^ Division of Pediatric and Adolescent Surgery University Hospitals of Geneva Geneva Switzerland; ^3^ Ministry of Health and Public Hygiene Ouagadougou Burkina Faso

**Keywords:** global surgery, pediatric

1

Historically, pediatric surgery has often been overlooked, largely because of perceptions regarding its high costs, complexity, and the extensive training it demands.^1^ As a result, efforts to address the gaps in pediatric surgery have frequently been fragmented, targeting isolated aspects rather than embracing a comprehensive approach.^2^ However, growing awareness has recently emphasized the need to strengthen entire health systems to advance pediatric surgery within the broader context of global health,^3^ highlighting key areas like training and staffing, infrastructure, quality and safety of care, research, and advocacy.^4^

Our unique program, titled *Pediatric Surgery Development Plan in Burkina Faso*, was designed to respond to the challenge of an integrated approach to tackle the pediatric surgical ecosystem in Burkina Faso, where children represent up to 45% of the population,^5^ promoting health system, strengthening principles, and targeting specific priority actions across all levels of the healthcare system. This program is the result of 5 years of close collaboration with the Ministry of Health, partner hospitals, and medical societies.

In Burkina Faso, despite limited resources for pediatric surgery, strong political will led to the establishment of a postgraduate diploma in pediatric surgery in 2017 and the deployment of pediatric surgeons across various regions. Additionally, the country has developed a National Strategy and Implementation Plan to bolster surgical and anesthetic care services.^6^ With this solid foundation, the environment was primed for the growth of pediatric surgery.

The program's areas of intervention, outlined in our *Pediatric Surgery Development Plan*, embody the global health philosophy mentioned earlier. They focus on six key areas: (i) infrastructure, equipment, and maintenance; (ii) quality of care; (iii) workforce development; (iv) community awareness; (v) research; and (vi) advocacy. Of note, the program's numerous interventions were supported through an official agreement between Geneva University Hospitals and the Burkina Faso Ministry of Health.

To strengthen the pediatric surgery infrastructure, we focused on the University Hospital Sourou Sanou in Bobo‐Dioulasso, the country's economic capital in the southwest. This choice aimed to relieve congestion in the centrally located capital Ouagadougou and reduce patient influx from the southwest to the center. Work included the rehabilitation of a pediatric operating room, a recovery room, and the construction of a new hospitalization wing, doubling the hospital's pediatric surgery capacity and enabling more complex treatments (situation 2018 vs. 2024: 28/44 beds; 1/5 pediatric surgeons; 8/23 ward nurses; 300/600 yearly operations; 814/1180 hospitalized patients). This hospital is now considered as the second national reference center for pediatric surgical care.

To standardize care for pediatric surgery patients and to ensure security of their management, in the country's two key university hospitals Ouagadougou and Bobo‐Dioulasso, quality tools and quality measures were implemented (the surgical checklist, gauze count in the operating room, structured pain assessment on the ward using the Evendol technique, regular monitoring of fluid balance, etc.). This included a clinical database with quality indicators (e.g., rates of surgical, swab count, and monitoring form) and the development of clinical protocols in surgical care.

Enhancing the training and skills of healthcare professionals in the country involved in pediatric surgical and anesthetic care was essential. In total, nearly one thousand professionals have been reached. For instance, we strengthened the advanced diploma in pediatric surgery by introducing specialized master classes on specific topics. Additionally, we provided the Safer Anesthesia From Education course (SAFE) to half of all national anesthesiologists and trained hundred anesthesia nurses in regional hospitals. We also developed specialized training modules for on‐site healthcare providers, including nurses and midwives, to enhance their skills. Finally, in collaboration with national public health schools, we integrated surgical care units into the academic curriculum to ensure comprehensive education for future nurses and midwives.

To raise community awareness, we launched two interventions specifically focused on addressing the high prevalence of domestic accidents in pilot regions, in collaboration with the Departments of Traditional Medicine and Health Education Promotion of the Ministry of Health. Training courses for traditional healers were created on open fractures and their early referral, attended by over 700 participants. The second intervention focused on preventing domestic accidents, which overload the country's pediatric surgical services, by training hundreds of community health workers to sensitize rural populations, reaching over 100,000 people. These two pilot projects have been designed to be replicable in all regions of the country and to be sustainably integrated into the Burkinabé landscape.

A selection of key indicators targeted within the Pediatric Surgery Development Plan in Burkina Faso is summarized in Table 1.

**TABLE 1:** Selection of key indicators targeted within the Pediatric Surgery Development Plan in Burkina Faso.

Indicators	Pre intervention (2018)	Post intervention (2024)	Target goal	% Achieved
N° of pediatric surgery referral hospitals in Burkina Faso	1	2	3	80%
N° of beds dedicated to pediatric surgery in these referral hospitals	81	97	117	83%
N° of surgeons enrolled in the advanced diploma in pediatric surgery	25	46	66^a^	69%
N° of anesthetists and nurse anesthetists trained in pediatric anesthesia in Burkina Faso	6	136	135	100%
N° of ward nurses specifically trained in pediatric surgery in Burkina Faso	0	350	500	70%
N° of midwives specifically trained in pediatric surgery in Burkina Faso	0	310	500	62%
N° of traditional healers trained in Burkina Faso's Grand Ouest region	0	700	1230^b^	56%
Number of people sensitized regarding domestic accidents in the Orodara health district	0	100,000	100,000^b^	100%

^a^ The Council of Ministers has approved the recruitment of 20 new pediatric surgeons as part of the advanced studies diploma for the year 2025. When this goal is achieved, there will be approximately one pediatric surgeon for every 150,000 children.

^b^ These are pilot regions, and the plan aims to integrate them into existing community and public health programs over the long term.

Of note, the various actions of this program have been supported by continuous data collection in collaboration with the Research Institute of Health Sciences in Ouagadougou. We conducted numerous research projects, which have helped to better direct our interventions. Additionally, the generation of scientific evidence bolstered our advocacy efforts.

Last but not least, a key objective of the program was to enhance the visibility and prioritization of pediatric surgery at the national level. By making the results of the program visible, conducting targeted research, and engaging with the country's scientific societies, we successfully established a robust advocacy framework. As a result of these efforts, the Council of Ministers approved scholarship grants for specialization in pediatric surgery awarded to 20 general surgeons. This strategy should be maintained for the next 2 years in order to equip the majority of hospitals in the country with pediatric surgeons. Another key focus of this advocacy effort will be the inclusion of pediatric indicators in the upcoming evaluation of Burkina Faso's national surgery and anesthesia strategy, following a study on the pediatric surgical capacity of hospitals nationwide.

In conclusion, the *Pediatric Surgery Development Plan in Burkina Faso* underscores the importance of a comprehensive approach to strengthening the surgical healthcare system. It has indeed resulted in significant and sustainable achievements through a strong collaboration rooted in a participatory approach, establishing a model that can inspire other low‐ and middle‐income countries in their efforts to enhance pediatric surgical care.

In the coming year, we will focus our efforts on reinforcing the sustainability of our actions. The continued support of the Ministry of Health fuels our commitment to strengthen the program's achievements and secure its long‐term sustainability, paving the way for a brighter future in surgical care for all children across the country.

## AUTHOR CONTRIBUTIONS

**Sophie Inglin**: Conceptualization; data curation; formal analysis; investigation; methodology; validation; writing—original draft; writing—review & editing. **Anata Bara**: Conceptualization; validation; visualization; writing—review & editing.

## ETHICS STATEMENT

Not applicable.

## THE GLOBAL CHILDREN'S SURGERY BURKINA FASO WRITING GROUP

Yacouba Traoré, Saïd N. Ganame, and Emile Bandre: Division of Pediatric Surgery, University Hospital of Sourou Sanou, Burkina Faso.

Abou Coulibaly, Seni Kouanda: Biomedical and Public Health Department, Research Institute of Health Sciences, Burkina Faso.

Bertille Ki: Division of Anesthesia and Intensive Care, Pediatric University Hospital of Charles de Gaulle, Burkina Faso.

Seydou Barro: General Direction, University Hospital of Sourou Sanou, Burkina Faso.

Karim Barro: Ministry of Health and Public Hygiene, Burkina Faso.

Barbara E. Wildhaber: Division of Pediatric and Adolescent Surgery, University Hospitals of Geneva, Switzerland.
